# Supplementary material for: Pro-Osteogenic and Anti-Inflammatory Synergistic Effect of Orthosilicic Acid, Vitamin K2, Curcumin, Polydatin and Quercetin Combination in Young and Senescent Bone Marrow-Derived Mesenchymal Stromal Cells
Source: Int J Mol Sci. 2023 May 16;24(10):8820. doi: 10.3390/ijms24108820 (PMC10218531; doi:10.3390/ijms24108820)
Supplement: Supplementary file 1 [file ijms-24-08820-s001.zip › ijms-2398209-supplementary.pdf]

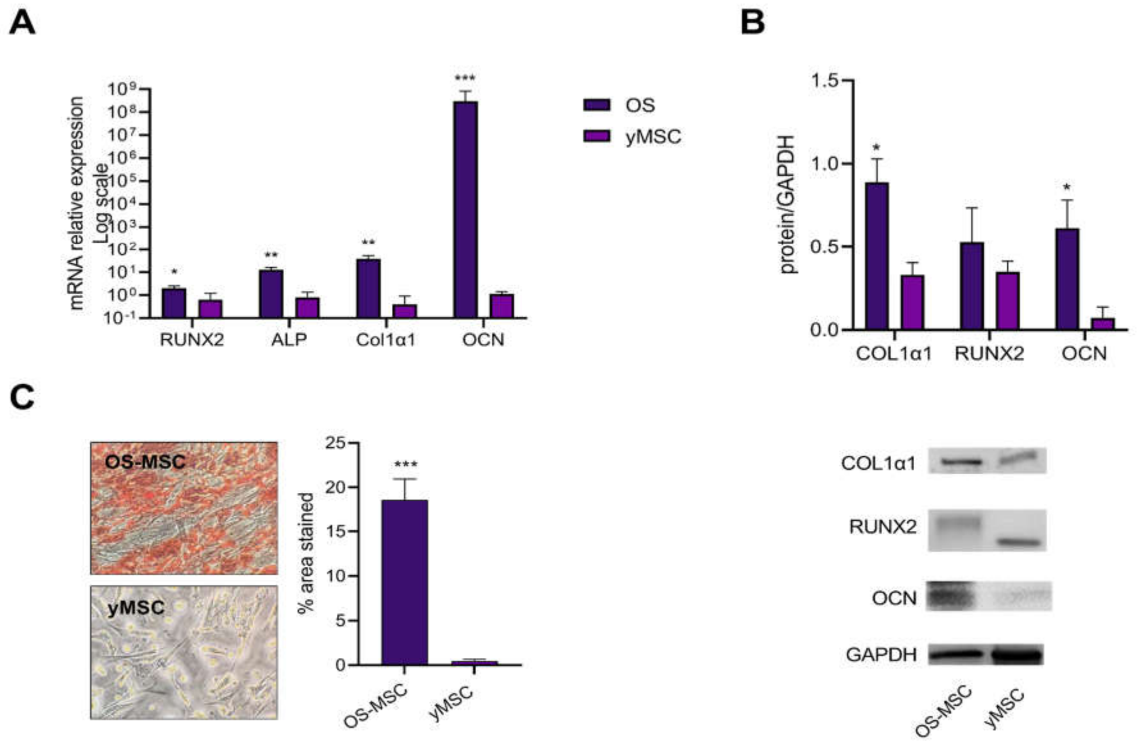

Figure S1. *Osteogenic differentiation of MSCs.* (**A**) RUNX2, ALP, Col1α1 and OCN mRNA expression of yMSC cultured in osteogenic (OS-MSC) or maintenance media for 7 days. Data are reported as relative expression according to $2^{-\Delta\Delta Ct}$ method, using IPO8 as housekeeping. (**B**) Representative western blot analysis of Col1α1, RUNX2 and OCN protein expression, with GAPDH as loading control. The bands were quantified with ImageJ. All data are reported as fold change vs untreated yMSC. The results are expressed as mean ±SD from three independent biological replicates. Paired t test, *$p<0.05$, **$p<0.01$, ***$p<0.001$ vs untreated yMSC. (**C**) Representative images of Alizarin red stained OS-MSC and yMSC. The histogram shows the mean of the percentage of area stained of three different photographs analysed with ImageJ.
